# Marine Plastic Waste in Construction: A Systematic Review of Applications in the Built Environment

**DOI:** 10.3390/polym17131729

**Published:** 2025-06-21

**Authors:** Lucas Lopes, Harish Dauari, Paulo Mendonça, Manuela Almeida

**Affiliations:** 1 Department of Civil Engineering, Institute for Sustainability and Innovation in Structural Engineering (ISISE), Advanced Production and Intelligent Systems (ARISE), University of Minho, 4800-058 Guimarães, Portugal; lucas_lopes@me.com; 2 Laboratory of Landscapes, Heritage and Territory (Lab2PT), School of Architecture, Art and Design, University of Minho, 4800-058 Guimarães, Portugal; harish.daruari@gmail.com (H.D.); mendonca@eaad.uminho.pt (P.M.)

**Keywords:** marine plastic litter, plastic waste in construction, sustainable built environment, plastic waste recycling

## Abstract

Marine plastic pollution represents a critical environmental challenge, with millions of tons of plastic waste entering the oceans annually and threatening ecosystems, biodiversity, and human health. This systematic review evaluates the current state of the art in recycling and reusing marine plastic waste within the architecture, engineering, and construction (AEC) sectors, following the PRISMA methodology. Sixty-six peer-reviewed articles published between 2015 and 2025 were analysed, focusing on the integration of plastic waste. The review identifies mechanical recycling as the predominant method, involving washing and shredding plastics into fibres or flakes for use in cementitious composites, asphalt modifiers, bricks, panels, and insulation. Results indicate that recycled plastics, such as PET, HDPE, and PP, can enhance thermal insulation, water resistance, and flexural strength in non-structural applications. However, challenges persist regarding compressive strength, fibre dispersion, and chemical compatibility with cementitious matrices. Although the reuse of marine plastics supports circular economy goals by diverting waste from oceans and landfills, significant gaps remain in long-term durability, microplastic release, end-of-life recyclability, and comprehensive environmental assessments. The findings underscore the need for further research on the broader adoption of life cycle analysis, as well as long-term durability and environmental contamination analyses.

## 1. Introduction

Plastic pollution has become a critical global environmental issue [1]. The damage caused by mismanaged plastic waste poses a significant threat to ecosystems, affecting organisms across all levels of the food chain [2]. It is estimated that between 4.8 and 12.7 million metric tons of plastic entered the oceans in 2010 alone [3]. Considering the continued growth in plastic production [4,5,6], a further increase in marine plastic pollution is anticipated. Plastic waste currently constitutes approximately 80% of all marine litter [7]. While some plastic remains buoyant, a significant portion ultimately sinks to the seabed [8].

Marine plastic pollution contaminates aquatic ecosystems with macroplastics, microplastics, and nanoplastics [9]. These pollutants not only harm marine life directly but also serve as vectors for persistent organic pollutants and heavy metals [10,11], posing serious threats to ecological balance and biodiversity. The primary sources of marine plastic include mismanaged plastic packaging—often transported by polluted rivers—and waste from the fishing industry [12,13]. Among the most frequently detected polymers in marine environments are polypropylene (PP), polyethene terephthalate (PET), and polyethene (PE), which together account for over half of all marine plastic debris [3,10]. For example, an analysis of the Han River found that PET was the most common microplastic type, indicating its widespread environmental distribution [14]. A broader meta-review of marine plastic pollution studies similarly identified low-density polyethene (LDPE), PP, polyvinyl chloride (PVC), polystyrene (PS), and PET as the five most frequently studied polymers, which also represent approximately 90% of global plastic production [10].

An increasingly relevant source of marine plastic waste is the improper disposal of surgical masks. The COVID-19 pandemic has significantly increased the presence of face masks in marine environments, contributing to pollution and ecological disruption. It is estimated that 25.9 ± 3.8 million metric tons of face masks entered the ocean in 2020 alone [15]. Although the proportion of surgical mask waste in marine ecosystems remains lower than that of packaging plastics, the rapid growth in this waste stream highlights a potential opportunity for diverting such materials into circular use.

Plastic waste in marine environments behaves differently depending on density and physical structure. Heavier plastics, such as PVC, PET, and polyamide (PA), tend to sink, while lighter polymers, like PE and PP, typically float [16]. However, buoyancy also depends on the condition of the material—for instance, containers with trapped air may float even if composed of denser plastics [17]. Degradation processes also influence this behaviour; for example, PP surgical masks may float initially but tend to sink after partial breakdown [18], while PET bottles may remain buoyant due to air pockets [19].

The degradation of plastics in the marine environment is influenced by several factors, including UV radiation, which accelerates deterioration, particularly in floating plastics [20]. Other contributing factors include hydrodynamic forces from water flow, mechanical wear from sand abrasion, and the corrosive action of saline conditions. These elements act synergistically to accelerate the fragmentation and surface degradation of plastic materials. Water flow can induce repetitive flexing and mechanical stress, while sand abrasion leads to surface pitting and micro-cracking. The saline environment further exacerbates polymer breakdown by promoting oxidative reactions and increasing brittleness, particularly in UV-aged plastics. Collectively, these factors significantly compromise the structural integrity and longevity of plastic debris in marine settings, facilitating the formation of microplastics and enhancing the leaching of additives into the environment [21]. This degradation complicates recycling, as the mechanical properties of the waste plastic are often degraded [22]. Despite these challenges, studies have demonstrated that recycling marine plastic remains feasible for specific downcycling applications. In the naval sector, for instance, recycled plastic has been used in 3D-printed parts for repairs and replacements [23].

Advancements in chemical recycling techniques, such as innovations in pyrolysis, also offer viable pathways for recovering value from degraded marine plastics [24].

Where recycling is not possible, energy recovery may be a more sustainable alternative to landfilling, particularly when the energy yield offsets the environmental cost of disposal [25]. In the construction sector, the use of plastic waste, especially packaging, is often employed in rotary cement kilns as an alternative fuel. This allows for the recovery of part of the energy spent on plastic manufacturing while ensuring the breakdown of the polymer, thereby diverting it from landfills and avoiding potential environmental contamination [26]. Such a process is monitored locally and regulated to ensure that plastic incineration does not compromise air quality and public health [27].

The widespread production of composites and multilayered materials has made the sorting and separation of plastic waste increasingly challenging. Due to the inherent incompatibility between different polymers, recycling without prior material sorting often leads to issues such as poor mechanical performance and brittle behaviour in the resulting products. However, recent studies have explored the use of additives to enhance the compatibility between certain polymers, potentially enabling a more efficient and cost-effective recycling process for mixed plastic waste [28,29].

In parallel with efforts to reduce marine pollution, the decarbonisation of the built environment has emerged as a global priority, particularly in alignment with 2050 climate targets [30,31]. The construction sector is responsible for approximately 37% of global CO_2_ emissions [32], making it a crucial target for emission reduction strategies. However, the decarbonisation of construction often requires significant inputs of materials and energy [33], which further increases the consumption of non-renewable resources [34].

The idea of integrating plastic waste into construction materials is not new. Some studies have investigated both unprocessed and mechanically recycled plastics in building applications. For example, unprocessed PET bottles have been used as structural containers for compacted earth, offering low-energy reuse solutions that reintegrate plastic into the economy while reducing emissions [35,36]. More advanced approaches involve mechanical recycling, where plastics are shredded, extruded, or moulded into components such as bricks, insulation panels, or additives for cementitious and bituminous materials [37,38]. These applications leverage the beneficial properties of plastic, including low weight, high strength-to-weight ratio, chemical resistance, and processability, while aiming to mitigate limitations, such as poor UV resistance and variable bonding performance, in cement matrices.

Despite the potential benefits, several challenges must be addressed to enable the feasible integration of plastic waste into the built environment. One of the most significant challenges is advancing the scientific understanding of plastic waste applications, particularly in terms of their performance from mechanical, cost, and long-term environmental perspectives. Concerns have been raised about the potential leaching of microplastics and nanoplastics into the environment, as well as contamination arising from polymer additives that may be released during the material’s life cycle [20,39].

Since marine plastic waste is subject to significant degradation due to prolonged exposure to environmental conditions, such as UV radiation, mechanical abrasion, and saline water, its recycling complicates the workability and mechanical performance of recycled marine plastics [8,40]. As a result, using marine plastic waste directly may present challenges related to poor bonding, brittleness, and reduced strength [41,42]. However, these degraded plastics can still be valuable in road construction, particularly as modifiers or binding materials in asphalt mixes [43,44]. Studies have shown that incorporating waste plastics into bituminous binders can enhance certain properties of asphalt, such as viscosity, elasticity, and resistance to deformation, although the workability of the mix will decrease according to the ratio of plastic used [45,46].

Additionally, regulatory frameworks and policy impacts present further challenges. Construction stakeholders may be influenced to adopt practices that incorporate plastic waste through targeted policies and incentives, which could also enhance public acceptance of these solutions [47,48]. In this topic, there are currently regulations regarding the use of recycled plastic. The European Union sets minimum recycled content requirements for plastic products. It supports the use of recycled plastics in public construction projects through directives, such as the Single-Use Plastics Directive [49,50]. India’s guidelines encourage the use of shredded plastic waste in road construction and co-processing in cement kilns, with clear procedures for collection, segregation, and utilisation [27]. These regulations are reinforced by public procurement policies and quality standards, collectively driving the adoption of recycled plastic waste in construction across various global contexts [51].

However, effective policy implementation requires further research and development, especially concerning the benefits and safety of integrating plastic waste into the construction sector [52]. Therefore, current research developments can support the identification of viable pathways for the circular use of plastic waste in construction while addressing issues related to safety and long-term environmental impacts.

To contribute to this goal, the present study conducts a systematic literature review of existing methods for recycling and reusing marine plastic waste in the architecture, engineering, and construction (AEC) sectors. By identifying the range of materials, recycling methods, and application areas explored to date, this review aims to support the integration of marine plastic waste into the built environment and promote the adoption of circular construction practices.

## 2. Materials and Methods

**Figure 1:**
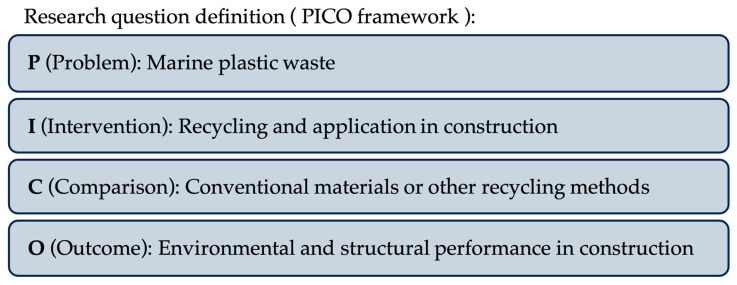
The PICO framework was used to define the research question addressed in this review.

Firstly, the research question was formed using the PICO framework, as shown in Figure 1.

The research question is “What are the current state-of-the-art recycling techniques for marine plastic waste and their applications in the built environment?”.

Secondly, the keywords for addressing the research question were selected revolving around plastic, waste, and construction. The relevant synonyms were selected as alternatives for searching queries. The results are presented in the cross-section of the three elements, as shown in Table 1. The number of keywords used was limited to a maximum of eight Boolean searches for the ScienceDirect database.

Thirdly, the database search was conducted in ScienceDirect and Scopus, using filters for review and research articles; the 10 years from 2015 to 2025; the English language; and the disciplines of Environmental Science, Materials Science, and Engineering. No registers were used. The search was conducted using the previously defined keywords in a Boolean logic using “OR” to separate each keyword from the same column and “AND” to connect the keywords during the timeframe of 9 March 2025 to 9 April 2025. The Boolean search query is shown in Table 2.

**Figure 2:**
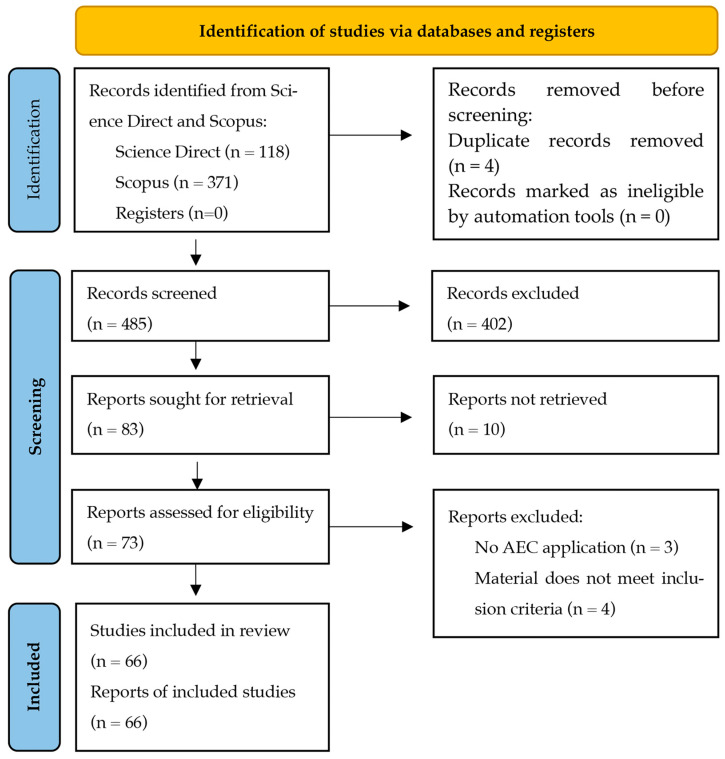
PRISMA flowchart of the review.

Lastly, the results were screened and documented in accordance with the PRISMA methodology, as illustrated in the flowchart presented in Figure 2. This review employed PRISMA as a qualitative systematic review, given the nature of the existing literature on marine plastic waste in construction, where most studies are qualitative or exploratory and do not lend themselves to quantitative meta-analysis. Therefore, PRISMA 2020 methodology was applied to ensure transparency and replicability in this review process.

The inclusion and exclusion criteria applied during the selection process are detailed below.

Inclusion Criteria

- Scope: Studies were included if they investigated the reuse, recycling, or valorisation of plastic waste originating from marine or coastal environments within the context of construction or the built environment. Alternatively, studies focusing on the recycling of single-use plastic waste commonly found in marine environments—specifically, light packaging and disposable surgical masks—were also considered, provided the recycled materials were applied in construction.
- Material type: The focus must include thermoplastics or thermosetting polymers (e.g., PET, HDPE, LDPE, PP, and PS).
- Publication type: Only peer-reviewed journal articles published in English were included.
- Publication date: Studies published between January 2015 and March 2025 were included.

Exclusion criteria:

- Studies unrelated to marine plastic waste or single-use plastics.
- Studies without explicit construction-related applications.
- Conference abstracts, editorials, patents, and grey literature.

No automation tools were used in the screening process. All articles were manually reviewed for eligibility, inclusion, and exclusion by the authors of this review. A protocol was not prepared for the review, and the search was not registered. This review did not include a formal risk of bias assessment due to the heterogeneous nature of the included studies and focus on material composition and applications. No specific method was used to assess certainty.

The PRISMA review identified 487 articles, of which 4 were duplicates and 10 were inaccessible at the time of the review. The review included only 66 articles, while the remainder was excluded according to the inclusion and exclusion criteria.

The included articles were reviewed to answer the research question. No additional data were sought beyond what does answer the research question. To improve the internal organisation and enhance the clarity of the review, the literature was categorised based on the source of waste: fishing industry waste, packaging, and surgical masks. Additionally, the review discusses recycling methods, associated challenges, and potential future developments.

## 3. Results and Discussion

This section presents and discusses the results of a systematic review of 66 peer-reviewed articles that explore the use of recycled plastic waste in construction. The review places particular emphasis on identifying waste types, prevailing applications, emerging opportunities, and research gaps in the context of marine plastic waste and its potential integration into the construction sector.

While not all selected studies directly address marine plastic waste, a significant portion involve recycling techniques applicable to marine litter, such as those used for disposable surgical masks and packaging-related plastics. Among the reviewed articles, 30 studies specifically address plastic waste from the marine environment or the fishing industry. The literature reviewed comprises experimental studies (*n* = 42), simulation-based research (*n* = 4), and literature reviews (*n* = 20).

Most studies aim to develop sustainable alternatives to conventional construction materials by incorporating recycled plastic waste into composite matrices, such as concrete, mortar, cement, and asphalt. These approaches often aim to enhance specific material properties, such as density, thermal insulation, and mechanical performance, while also addressing the broader environmental benefits of recycling plastic waste.

**Figure 3:**
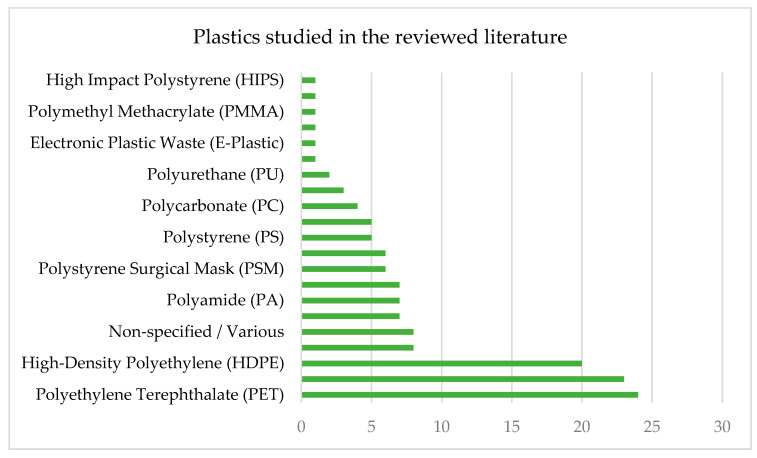
The frequency of different plastic materials identified in the reviewed literature shows a focus on PET, PP, HDPE, and LDPE.

Among the polymers studied, polyethene terephthalate (PET), high-density polyethene (HDPE), and polypropylene (PP) were identified as the most applied materials across a wide range of construction applications. Figure 3 illustrates the frequency of various plastic materials in the literature, highlighting the most commonly reused polymers, including HDPE, PET, and PP.

**Figure 4:**
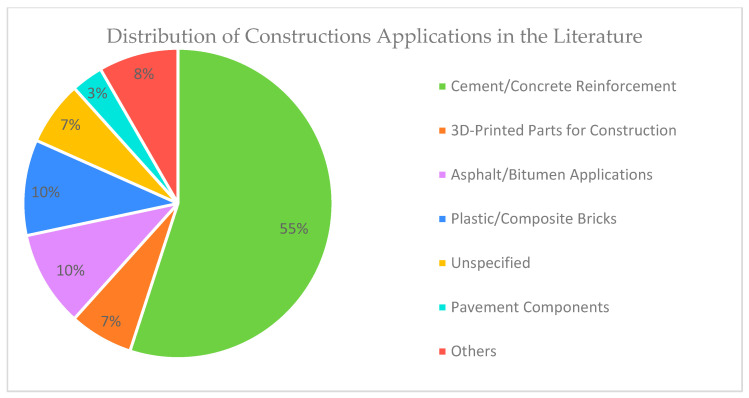
Distribution of construction-related applications for recycled plastic waste.

Figure 4 shows the distribution of construction-related applications for recycled plastic waste, where the definitions of each use are shown in Table 3, where the results point towards heavy usage of plastic waste in cementitious and bituminous matrices, ranging from fibre reinforcement to binder, with a small amount of the literature focusing on applications such as wood–plastic panels, gypsum reinforcement, and compressed earth block additives.

### 3.1. Fishing Industry Waste

Plastic waste from the fishing industry is a major contributor to marine pollution, yet its reuse in construction remains underexplored. Existing studies primarily focus on recycling HDPE, LDPE, PP, and PA from fishing nets, lines, and gear for use in cementitious composites.

#### 3.1.1. Fishing Waste in Cementitious and Bituminous Composites

Recycled fishing plastics are commonly used as fibres in concrete and mortar to enhance tensile and flexural properties. Truong et al. [53,54] employed whole, epoxy-coated nets in seawall and mortar layers, noting significant preparation challenges. More commonly, shredded fibres are added to mortar and concrete, as in Park et al. [55], who reported improved flexural toughness and compressive strength (up to 47.47 MPa), despite clumping and uneven dispersion. X-ray tomography by Pae et al. [56] confirmed increased porosity and fibre clustering, enhancing flexural but reducing compressive strength.

Though a trade-off between tensile and compressive strength was noted, Nguyen et al. [57] emphasised corrosion resistance and marine suitability. Hussan et al. [58,59] proposed low-cost cleaning via rainfall and subsequent NaOH treatment, improving chemical compatibility with cement.

Overall, fishing net fibres improve flexural strength, crack resistance, and durability, though challenges in processing, dispersion, and bonding persist, especially at higher dosages.

#### 3.1.2. FRP Solutions and 3D Printing

Gopinath et al. [60] used recycled PE braided ropes in FRP composites with epoxy resin, enhancing flexural performance while highlighting the need for epoxy-free alternatives. Lopes et al. [40] reprocessed degraded HDPE mooring cables into filaments for 3D printing, validating feasibility but noting uncertain UV and fire resistance. Kyriakidis et al. [61] concluded that such applications suit non-structural elements due to limited mechanical performance.

In summary, plastics in the fishing industry offer promising construction applications, particularly in cement-based systems. However, processing, durability, and scalability challenges remain, highlighting the need for further real-world testing and standardisation. Plastic waste from the fishing industry is often cited as a significant contributor to marine pollution. Although plastics are widely used across various fishing-related activities, few studies have examined their recycling and reuse, specifically within the construction sector. The available literature primarily focuses on recycled high-density polyethene (HDPE), low-density polyethene (LDPE), polypropylene (PP), and polyamide (PA) from sources such as fishing nets, lines, gear, and mooring cables.

### 3.2. Plastic Packaging Waste

Single-use packaging plastics (e.g., PET, HDPE, and LDPE) are significant pollutants that often enter marine environments through improper disposal. Due to their abundance and environmental relevance, they are widely studied for use in construction applications.

#### 3.2.1. Packaging Plastics in Cementitious and Bituminous Composites

These plastics are commonly recycled mechanically and used as aggregate replacements or reinforcement fibres in cementitious materials. Moderate additions (10–15%) can retain or improve mechanical properties, while higher contents typically reduce strength due to poor bonding and porosity [36,45,62].

Shredded HDPE and LDPE improved flexural strength and impact resistance, though dispersion issues persisted [55,63]. PET showed promise as a fine aggregate [64,65] and as a binder [66], but ocean-derived PET required more cement to offset strength losses [65].

Studies also reported reduced strength when PET and HDPE were used as sand-like aggregates, stressing the need for surface treatment [67,68]. Nonetheless, PET-based mortars were shown to be non-toxic [69]. In road applications, recycled PET has been shown to enhance asphalt performance in hot climates [70].

#### 3.2.2. Minimal Processing and Alternative Applications

Low-processing approaches included PET bottles filled with sand or soil as wall units, offering compressive strength that is similar to traditional bricks [65,71].

#### 3.2.3. Use in Clay and Cement Bricks

Plastics were used in brick production, with HDPE bricks showing good thermal and mechanical performance [72]. However, poor bonding often leads to increased porosity and cracking [73]. PET additives met local standards in earth–cement bricks [74], while PET and HDPE improved insulation in clay bricks but reduced strength at high contents [75].

#### 3.2.4. Non-Cementitious and Composite Applications

Fully plastic bricks (HDPE and PP) performed better but required surface modifications for bonding [76,77].

#### 3.2.5. Structural Applications

While typically suited for non-structural use, PET-reinforced beams showed slight strength gains [78]. PET-sand composites also demonstrated potential as binder replacements in low-strength structural contexts [79].

In conclusion, packaging plastics offers viable pathways for integration into construction materials, supporting circular economy goals.

### 3.3. Surgical Masks

The COVID-19 pandemic led to a surge in disposable surgical mask waste, much of which enters marine environments. Composed mainly of PP fibres, these masks have recently been explored for reuse in construction, particularly in cementitious composites and asphalt.

Several studies examined the incorporation of shredded masks into mortar and concrete [80,81,82,83]. Small additions generally improved flexural strength and crack resistance, though slight reductions in compressive strength were reported [80]. Common challenges included fibre clumping, porosity, and uneven distribution [81]. Proper disinfection, microplastic release, and toxicity assessment are essential [82].

In asphalt, Yalcin et al. [84] found that 1–3% ground mask fibres improved rutting resistance and stiffness at high temperatures, though further investigation into durability and fire resistance was recommended.

Thoudam et al. [85] integrated mask fibres into alkali-activated formulations for bricks, achieving acceptable compressive strength, reduced weight, and lower water absorption. However, concerns about polymer reactivity under alkaline conditions were noted.

While mask fibres enhance ductility and energy absorption in composites, the absence of standardised processing and testing remains a key limitation. Overall, reusing surgical mask waste presents opportunities for waste reduction and material improvement, particularly in non-structural applications, but further research is needed on safety, dispersion, and large-scale feasibility.

### 3.4. Pre-Processing Techniques and Recycling Methods

Mechanical recycling is the most common method for incorporating plastic waste into construction materials, favoured for its simplicity, low cost, and scalability. Other recycling methods were beyond the scope of this review, representing a noted limitation.

Most studies begin with waste collection and manual or mechanical sorting, followed by washing and drying, critical steps for ensuring material quality [40]. Sorting plastic waste is a challenging task. Not all articles address sorting methods, and the ones that do point out this area as an opportunity for improvement [7,9]. On the other hand, some of the literature focuses on the recycling of plastic without sorting by material, which often results in suboptimal mechanical performance, albeit with the trade-off of power costs and emissions [41,78,86].

Plastics are then shredded or ground into flakes or fibres to enable integration into cementitious or bituminous matrices or for thermal processing. These, in some cases, are again sorted by flake size or fibre length, which does influence the performance of the construction material integrated with the plastic waste [45,86].

To enhance compatibility with cement, surface treatments such as sanding [87], NaOH immersion [85], and thermal conditioning [66] are employed, addressing persistent issues with interfacial bonding. For thermoplastics used in bricks or panels, melting and moulding via hot pressing or extrusion—typically around 300 °C—has yielded improved mechanical and thermal performance [46,77]. Some studies have also produced filaments or pellets for use in 3D-printing construction elements [40].

Low-energy pre-treatment methods, like natural rainfall cleaning of fishing nets, have also been tested, offering solutions for low-resource contexts [58,59].

While mechanical processing is widely adopted and adaptable, challenges remain in fibre dispersion, size control, bonding, and scalability. Further optimisation is needed to support real-world applications.

### 3.5. Applications in Construction

The reviewed studies highlight a growing interest in incorporating recycled plastic waste into various construction applications, ranging from non-structural to structural uses. The suitability of these applications depends on the type of plastic, the processing technique, and the physical and mechanical requirements of the intended use.

#### 3.5.1. Overall Non-Structural Applications

Most studies focus on non-structural applications, where the technical performance demands are lower, and plastic waste integration is more feasible. Abubakar et al. [65], Dadzie et al. [71], and Kulkarni et al. [77], for example, demonstrated that plastic bricks, comprising partially or fully melted HDPE or PET, can achieve compressive strengths comparable to or exceeding those of conventional clay bricks. These materials also exhibit reduced density, improved thermal insulation, and lower water absorption. Additionally, studies indicate that plastic-based concrete demonstrates enhanced chloride ion resistance due to the hydrophobic nature of polymers such as PET and HDPE [54,86]. This property makes it particularly suitable for coastal infrastructure and marine applications where corrosion resistance is necessary.

Likewise, shredded plastic fibres (e.g., from packaging or fishing gear) have been shown to improve flexural strength and crack resistance in cementitious composites used for pavements, façade panels, and walkways. Nonetheless, trade-offs such as reduced workability, clumping, and inconsistent bonding at the plastic–cement interface, especially at higher plastic dosages, are frequently reported.

#### 3.5.2. Overall Structural Applications

Fewer studies address the application of recycled plastic waste in structural elements, and those that do report mixed results. Research by Jesus et al. [78] and Chong and Shi [64] examined PET and HDPE in concrete beams and columns, noting slight gains in flexural or tensile strength in some cases. However, many studies have found these improvements to be statistically insignificant. Generally, recycled plastics can contribute to ductility and toughness but often at the cost of compressive strength, particularly when added without surface treatments or in coarse forms.

Some promising findings relate to semi-structural applications. For example, Truong et al. [53,54] found that fishing net reinforcements improved mechanical performance in seawall panels and cement–mortar interfaces, although they required laborious preparation. Similarly, when optimally sized and dosed, plastic aggregates can achieve mechanical thresholds suitable for light structural components, such as low-rise walls or temporary infrastructure.

#### 3.5.3. Trade-Offs

Across both structural and non-structural applications, performance is strongly influenced by particle size, fibre shape and orientation, dispersion quality, plastic-to-binder ratio, surface texture, and any chemical pre-treatment.

While plastic inclusion often enhances thermal insulation, water resistance, and flexural strength, it also introduces porosity, lowers compressive strength, and complicates workability. However, the improved chloride resistance of plastic-modified concrete offers a distinct advantage in marine environments, potentially extending the service life of coastal structures by mitigating the corrosion of steel reinforcement [54,86]. These trade-offs, as summarised in Table 4, underscore the need for application-specific optimisation, with non-structural uses remaining the most practical entry point for widespread adoption.

### 3.6. Environmental Benefits and Challenges

Environmental motivations underpin much of the literature, linking the use of recycled plastic in construction to circular economy, waste reduction, and carbon mitigation goals.

A key benefit is the diversion of plastic waste—particularly, PET, HDPE, and LDPE—from landfills and marine environments, extending material life cycles and reducing leakage. Substitution of natural aggregates [55,69] and potential reductions in cement use contribute to lower carbon emissions. Additionally, lightweight composites with low thermal conductivity may improve building energy efficiency [35,88].

Mechanical shredding and low-temperature moulding are less energy-intensive than traditional recycling. Chen et al. [89] demonstrated that dual-use PET processing (asphalt modifiers and biodiesel precursors) offers environmental advantages without hazardous additives.

However, critical challenges persist. The risk of microplastic release and lack of data on long-term degradation remain major concerns, especially in marine or high-moisture contexts. Ocean-sourced PET, for instance, requires more cement to maintain its strength, thereby increasing emissions and costs [86].

End-of-life recyclability is also problematic. Plastics embedded in cement or hybrid composites are challenging to separate and reuse, which raises concerns about future waste management issues [76]. Moreover, few studies include life cycle assessments, toxicity analyses, or exposure assessments, which limits environmental evaluation [72].

In sum, while recycled plastics offer clear environmental benefits, further research is needed on long-term durability, recyclability, and ecological impact. Life cycle and GHG assessments remain notably underutilised in the current literature.

### 3.7. Policy and Practice Implications

Several studies in the reviewed literature emphasise the need for policy intervention to support the adoption and regulation of marine plastic waste in the construction sector. While specific policy proposals are often absent, the literature highlights the importance of regulating material applications, defining acceptable proportions of plastic waste in composites like concrete, and standardising recycling and integration procedures.

To advance the use of marine plastic waste in construction, comprehensive policy measures and industry strategies are essential. Governments should develop regulatory frameworks that encourage the use of recycled marine plastics, especially in non-structural applications, such as insulation panels, pavers, and formwork. These frameworks should include quality standards, performance benchmarks, and safety criteria to ensure market acceptance and public trust [49].

Expanding extended producer responsibility (EPR) schemes to cover marine plastic recovery can provide financial incentives for integrating recovered waste into building materials. Public procurement policies should also prioritise projects that use recycled marine plastics, supporting demand and supply chain development [90].

Additionally, research funding should focus on assessing the long-term durability, microplastic release, and recyclability of these materials to ensure that the environmental benefits outweigh the potential risks [91]. Cross-sector collaboration among industry stakeholders, waste management entities, and researchers, along with educational initiatives, will further enable the responsible adoption of marine plastic-based materials in construction.

### 3.8. Research Gaps and Future Directions

Despite growing research on the use of recycled plastics in construction, key gaps still hinder their full environmental, technical, and economic potential. The most cited limitation is the lack of long-term durability assessments. While mechanical performance is often reported under controlled conditions, real-world factors, such as UV exposure, freeze–thaw cycles, moisture, and chemical resistance, are rarely tested in these conditions. These are crucial for evaluating microplastic release, embrittlement, and material stability in outdoor or marine settings.

Additionally, there may be a risk of reporting bias, as studies with positive outcomes related to plastic reuse in construction are more likely to be published. Although possible negative impacts are mentioned, primarily in terms of toxicity and microplastic pollution, there is a gap in further exploring these impacts and addressing potential biases that could distort the overall understanding of plastic waste recycling in construction.

Environmental analyses are similarly limited. Few studies include life cycle assessments, and even fewer address toxicity, leaching, or occupational health risks, especially for thermally processed or non-recyclable composites.

Technically, challenges persist in fibre dispersion, orientation, and bonding. Clumping, weak interfacial adhesion, and uneven distribution reduce performance and workability. Surface treatments, coupling agents, and bio-based binders merit further study.

Economically, most efforts remain at the lab or pilot scale. Few explore scalability, supply chain integration, or logistical challenges in low-resource settings. Research on cost-effectiveness, infrastructure compatibility, and labour demands is still lacking.

## 4. Conclusions

In this systematic review, 66 recent literature papers on practices for recycling and using marine plastic waste in construction have been summarised. A meta-analysis was not feasible due to data heterogeneity, the lack of effect estimates, and the nature of the studies, which were primarily qualitative or exploratory. Nevertheless, the qualitative results are clear and answer the research question.

Mechanical recycling methods are the most common approaches, using simple mechanical procedures such as washing and shredding. The inclusion of plastics in cementitious and bituminous matrices was the predominant application of the recycling of such waste in construction. The addition of recycled plastics in construction elements has been reported to be a trade-off, offering improved flexural strength, lightness, and water resistance but at the expense of decreased compressive strength and workability.

Several challenges remain. Environmental concerns, including the release of microplastics, long-term durability, and recyclability, remain largely unaddressed. There is also a lack of comprehensive life cycle assessments and environmental impact studies, which limits a full understanding of the sustainability of these practices.

Further research into experimental performance and durability, especially in pilot projects, is necessary for future development. Policies regulating procedures and performance while providing incentives for stakeholders are essential for the broad adoption of plastic waste in construction.

Using marine plastic waste in construction offers potential benefits for the circular economy and pollution reduction. However, important research gaps must be addressed. Future work should focus on improving material formulations, strengthening the bond between plastics and construction matrices, and evaluating the long-term performance and environmental safety of these materials. Pilot projects and field studies will be crucial for testing these solutions in real-world conditions.

Overall, the responsible use of marine plastic waste in construction can contribute significantly to the recycling and diversion of plastic waste. However, the feasibility of such recycling applications will depend on further research, effective regulations, and clear industry standards.

## Figures and Tables

**Table 1:** Research entries used in the query for this review.

Keyword 1	Keyword 2	Keyword 3	Keyword 4
Ocean plastic	Waste	Recycling	Construction
Marine plastic	Litter	Reuse	
	Pollution		

**Table 2:** Boolean query used for the literature search based on the defined keywords in Table 1.

Boolean Search Query
(“marine plastic” OR “ocean plastic”) AND (“waste” OR “litter” OR “pollution”) AND (“recycling” OR “reuse”) AND (“construction” OR “built environment”) AND (LIMIT-TO (SUBJAREA, “ENGI”) OR LIMIT-TO (SUBJAREA, “MATE”)) AND (LIMIT-TO (DOCTYPE, “ar”) OR LIMIT-TO (DOCTYPE, “re”) OR LIMIT-TO (DOCTYPE, “cp”)) AND (LIMIT-TO (PUBSTAGE, “final”)) AND (LIMIT-TO (LANGUAGE, “English”))

**Table 3:** Construction applications and categories are shown in Figure 4.

Category	Applications
Cement/Concrete Reinforcement	Cement and/or concrete reinforcement
	3D-printed concrete reinforcement
	Cement road pavement
	Concrete binder and/or additives
	Plastic sand mix
	Concrete interface matrix
3D-Printed Parts for Construction	3D-printed parts (excluding explicitly stated as reinforcement)
Asphalt/Bitumen Applications	Asphalt and bitumen additive
	Bitumen binder
Plastic/Composite Bricks	Plastic bricks
	Concrete brick reinforcement
	Soil cement bricks additive
	Clay brick additive
	Earth bricks additive
Others	Thermal insulation
	Gypsum reinforcement
	Railway infrastructure
	Wood–plastic mixture
	Carbon fibre-reinforced polymer panels
Pavement Components	Pavement slabs and blocks
Unspecified	Unspecified

**Table 4:** Summary table highlighting the benefits and trade-offs identified in this literature review, pointing out opportunities for future research.

Benefits	Trade-Offs
Reduces landfill and marine pollution	Collection and sorting of plastic waste can be complex and costly
Lowers construction material costs	Mechanical properties (e.g., compressive strength) may decrease, especially at high plastic contents
Enhances thermal insulation and water resistance	Workability and bonding with cementitious matrices can be problematic
Improves flexural strength and crack resistance	Increased porosity and risk of microplastic release
Supports circular economy and resource conservation	Long-term durability and environmental safety remain uncertain
Lightweight and easy to mould into various forms	Susceptibility to UV degradation and flammability concerns
Can be used in non-structural and some structural applications	Not suitable for high-load structural elements without modifications
Energy savings compared to producing virgin materials	The end-of-life recyclability of plastic-infused composites is limited
Provides new ways for waste management (e.g., masks and fishing nets)	Potential health and safety risks for workers and occupants
